# Addressing Africa’s Energy Dilemma

**DOI:** 10.1007/978-3-030-39066-2_7

**Published:** 2020-03-26

**Authors:** Lapo Pistelli

**Affiliations:** 1 grid.16989.3f 0000 0004 1757 6313 Fondazione Eni Enrico Mattei, Milan, Italy; 2 grid.16989.3f 0000 0004 1757 6313 Fondazione Eni Enrico Mattei, Milan, Italy; grid.423791.a 0000 0004 1761 7437 Eni, Rome, Italy

## Abstract

This chapter discusses how the ongoing low-carbon energy transformation could reshape geopolitics within Africa and between the continent and the rest of the world. The chapter first attempts to define what ‘transition’ means in African contexts and if the concept applies at all to African dynamics. It then delves into the drivers and modalities of Africa’s alleged shift to finally explore geopolitical dynamics, questioning whether Africa is still the locus for the global supply of natural resources, introducing patterns of engagement between Africa and international/regional actors, and finally presenting the socio-economic implications of the shift. We conclude that while the *venues and sources* of geopolitical interest might change in the new geopolitical order that the transition to renewable energy implies, the *content and modalities* of interaction may see a continuity with the past, namely, dependence on external financing and technology. With yet at least one novelty: increased relevance of regional interdependencies.

## Introduction

The transition to cleaner forms of energy production is happening in sub-Saharan Africa (SSA) with distinct characteristics compared to other world regions. African countries are gifted with a huge—and still largely untapped—energy potential but energy access is among the lowest in the world, mainly due to structural constraints (such as the poor efficiency of the power sector and the non-capillary diffusion of grids) as well as lack of significant investment in energy infrastructure, human resources and technology.

Considering that energy is the main enabler of economic development and an essential component of several Sustainable Development Goals (SDGs), if universal energy access in sub-Saharan Africa continues to be a distant objective, a large part of the 2030 Agenda for Sustainable Development will remain out of reach, depriving the region of the possibility to build a prosperous future. Thus, guaranteeing energy access is *the* priority of sub-Saharan countries, while the transition from fossil fuels to cleaner forms of energy is *a* complementary priority that manifests itself very differently across countries in terms of timing, modality and impact.

This chapter discusses how the ongoing low-carbon energy transformation could reshape geopolitics within Africa and between the continent and the rest of the world. The chapter first attempts to define what ‘transition’ means in African contexts and if the concept applies at all to African dynamics. It then delves into the drivers and modalities of Africa’s alleged shift to finally explore geopolitical dynamics, questioning whether Africa is still the locus for the global supply of natural resources, introducing patterns of engagement between Africa and international/regional actors, and finally presenting the socio-economic implications of the shift.

The analysis pivots between discussions of contemporary developments and future trends. The former accounts inevitably differ from those that elaborate on the ‘likely’ implications of renewables on geopolitics in the coming decades. While the study of current settings is based on historical and factual accounts, investigations of ‘likely’ futures are based on assumptions and scenarios.

We conclude that while the *venues and sources* of geopolitical interest might change in the new geopolitical order that the transition to renewable energy implies, the *content and modalities* of interaction may see a continuity with the past, namely, dependence on external financing and technology. With yet at least one novelty: increased relevance of regional interdependencies.

These latter may signal a counter-tendency at a time when increased tariffs and sanctions are everyday practice at the global level. The recent signing of the landmark African Continental Free Trade Area (AfCFTA) agreement in July 2019 emphasises Africa’s willingness to reduce reliance over external partners and products while increasing regional integration. It is, however, a trend that has yet to materialise while it already presents pitfalls. According to the United Nations Conference on Trade and Development (UNCTAD), increased intra-African trade will depend, among others, on how ‘rules of origin’^1^ will be negotiated and implemented. Unless such rules are well thought through, future regional value chains on the continent will continue receiving little input from Africa, and a lot from abroad.

Importantly, at the time of revision of this book chapter, the world is experiencing an unprecedented pandemic, unforeseeable at the time of writing. The global economy is headed for recession and the impact on the African continent is very likely to be severe with potential social, economic and political disruptions. However, although investments and new electricity connections will slow down as a result of the crisis, our analysis and conclusions have so far remained solid: the pandemic will not reduce the urgency for universal energy access whose achievement underpins the success of the Sustainable Development Agenda; global lockdowns have highlighted the critical value of power infrastructure and this applies also to Africa, especially when it comes to the development of regional energy markets; the role of clean energy sources will continue to be strategic thanks to the crescent competitiveness of renewables and the complementary role of natural gas; furthermore, if there is one thing that the coronavirus pandemic has once again confirmed is the reliance of Africa on external actors, and yet its increasing willingness and capacity to coordinate at the continental and sub-continental levels.

## Understanding the Energy Transition in Sub-Saharan Africa

When we talk about the energy transition in sub-Saharan Africa, we cannot avoid problematising the concept of ‘transition’, which generally implies moving from a socio-economic and technological arrangement to another. The assumption behind the concept is that an arrangement does indeed exist. In sub-Saharan Africa, however, 600 million people (IEA 2018), over a total 1 billion (World Bank 2018a),^2^ lack energy access. The ‘transition’ to cleaner forms of energy is thus only a complementary concern, second to that of providing access to energy. In other words, if the majority of the population does not have access to energy, it does not have anything to transit away from.

Nonetheless, as governments conceive national development plans, the idea of leapfrogging the conventional path to energy development and shift directly to renewables is starting to be contemplated. The ‘National Determined Contributions’ (NDCs)^3^ to the Paris Agreement and the energy plans of many sub-Saharan countries indicate an initial move towards the energy transition. Differences are, however, great across the continent. Countries like Kenya and South Africa have commissioned a significant number of renewable energy projects in the last years (African Energy Live Data 2019),^4^ though the inefficiencies of the power sector and the inadequacy of the energy infrastructure (common structural constraints in the sub-Saharan context) currently make the substantial penetration of additional capacity a tough challenge. However, the vast majority of sub-Saharan countries are still very far from expanding the share of renewables in the power mix.

Biomass and waste^5^ cover about 60% of sub-Saharan Africa’s *energy* mix, followed by oil and coal (16% each), gas (4%), nuclear and hydro (1% each), while renewables combined represent a mere 1% (Enerdata 2019). With a 51% share in the *electricity* mix,^6^ coal is the dominant fuel in electricity production.^7^ Gas holds 11% of the share, oil 7%, hydro 24% and solar, wind and geothermal combined 3% of electricity generation (Enerdata 2019). On average then, renewable energy in sub-Saharan Africa represents a mere 1% reality—yet with revolutionary potential.

### Energy Access Is the Priority of the Region

Africa’s energy landscape is extremely diverse across the continent. It presents different energy structures (based on reliance on various natural resources), different levels of infrastructure development and consequential varied vested interests in specific sources, such as oil in Nigeria and Angola, coal in southern Africa. Nonetheless, a distinctive feature shared by all sub-Saharan countries is the world’s lowest rate of energy access.

Although the overall electrification rate in sub-Saharan Africa has ‘almost doubled since 2000, rising by 20% points to 43%’ (IEA 2018, p. 78), today six people out of ten remain without electricity and have no promising prospects for improving their conditions any time soon. The sub-continent is still home to 20 countries with the world’s lowest electrification rates and almost nine people out of ten (890 million over a billion) have no clean cooking access while the number of people relying on biomass, coal and kerosene for their main household cooking needs has increased by 270 million since 2000 (IEA 2018). Moreover, progress on energy access, uneven as it has been across the continent, concentrated in just four countries—Kenya, Ethiopia, Tanzania and Nigeria—which make up more than half of those gaining access since 2011.

This is particularly problematic not just for the achievement of Sustainable Development Goal #7, but also for the achievement of several other development goals, given that energy is the main enabler of economic development. It is estimated that if universal energy access in sub-Saharan Africa continues to be a distant objective, most of the 2030 Agenda for Sustainable Development will remain out of reach (IEA, IRENA, United Nations Statistics Division, World Bank, World Health Organization [WHO] 2018), subsequently depriving the region of the possibility to build a prosperous future.

To revert the trend and ensure universal energy access, the International Energy Agency foresees that growing electricity needs in sub-Saharan Africa will have to be met by using a combination of domestic gas and renewable energy sources, reducing as much as possible the use of oil and coal in power generation. Also, the use of biomass will have to be substituted with improved biomass cookstoves and Liquefied Petroleum Gas (LPG) as clean cooking options.

Considering that clean cooking represents only a minor part of the incremental investment required to ensure universal energy access in Africa, this article will mainly focus on the power sector, looking at the substitution of coal and oil with natural gas and renewables. These latter two resources are widely available in Africa, and the economy of the region would very much benefit from their increased use for power generation.

### Structural Constraints to Gas and Renewable Energy Penetration in the Power Mix

Increasing the use of natural gas and renewables for power generation is not, unfortunately, easily achievable, though desirable, in sub-Saharan Africa. Highly efficient and economically sustainable power systems, together with costly infrastructure investments, are required to ensure the substantial penetration of those resources in the electricity mix. The absence of these two conditions across the continent constitutes the main reason why sub-Saharan power markets are underdeveloped, and gas-to-power and renewable energy projects are still relatively limited in the region. In many cases, this is also a problem for the oil and gas sector, where the development of gas projects are, in many cases, conditioned to the availability of liquefaction facilities for the sale of gas in the international gas markets.^8^

There is, however, one more underlying reason as to why the power sector faces challenges across the continent: the lack of industrial capacity, which leads to relatively limited energy demand, and hence the uselessness of increased power generation.

#### Efficiency and Economic Sustainability

Concerning efficiency and economic sustainability, a study of the World Bank (Kojima and Trimble 2016) analysed the relationship between costs and revenues of electric utilities in 39 sub-Saharan countries highlighting that only Uganda and Seychelles fully recover their operational and capital costs with the cash collected from customers. Such large deficits^9^—averaging 1.5% of gross domestic product (GDP)—‘prevent power sectors from delivering reliable electricity to existing customers, let alone expanding supply to new consumers at an optimal pace’ (Kojima and Trimble 2016, p. vii). The commercial losses faced by utilities raise the issue of ‘creditworthiness’: the deficit puts national utilities under financial stress—also affecting public finances since power utilities are usually state-owned entities—and exacerbates the off-take risk.^10^ This sub-optimal situation hinders private investments in power generation even where the power grid would allow additional generation capacity to reach the final customer.

#### Infrastructure Investment

However, power generation is only one part of the electricity sector value chain, and one of the most critical elements of sub-Saharan Africa’s energy sector is indeed related to the development of the power grid. The inadequacy of the transmission and distribution network prevents the energy supply from reaching final demand representing one of the main causes of the low levels of energy access in the region.

While in the oil and gas industry the possibility to export and sell the resource on international markets constitutes a sufficient incentive for the private sector to invest, investments in electricity generation can only be paid back by the local (or regional) market. Hence, a well-functioning power grid is essential to attract private investments.

The ‘natural monopoly’ characteristics of transmission and distribution make the intervention of the public sector necessary, but the budget constraints suffered by the vast majority of sub-Saharan countries limit the scale of their intervention. For gas-to-power projects, the challenge is even tougher than renewables since the former requires infrastructure investments also in the midstream sector for the provision of gas-to-power power plants.^11^

#### Lack of Industrial Capacity

Though access to energy is certainly a necessary condition for economic development, economic development is, in turn, necessary to increase energy demand. A country that has limited industrial capacity is likely to need a relatively limited quantity of electricity.

Ghana, for example, has a rather strong economy if compared to the sub-Saharan region, and has made considerable gains in the expansion of energy access in recent years—providing 79% of the population with electricity in 2017 (World Bank 2018b). After suffering power shortages in 2014 and 2015, the government reactivated fast-tracking private power plants. However, today the country faces the opposite problem: excess electricity.^12^ In other words, demand is lower than supply, posing severe financial risks to Ghana’s economy. One might well ask why the power supply does not automatically generate its own demand among the 79% of the population already connected to electricity—especially considering the low GDP^13^ per capita of the country (World Bank 2019a)—but energy supply will never ensure industrial development if adequate policy measures are not adopted by the government. In other words, energy access is a necessary—but not sufficient—condition to industrial development. The case of Ghana well represents a situation in which limited growth in energy demand does not depend on limited supply but on macro-economic constraints.

As of now, there are only a few countries in the sub-Saharan region where industrial development could drive energy demand growth. South Africa, Kenya and Ethiopia represent different examples of this. South Africa is the most advanced economy in the continent, but its industrial production growth is hindered by frequent electricity shortages. In Kenya, with the blueprint development programme ‘Kenya Vision 2030’, the government aims to achieve middle-income status by 2030, continuing an extraordinary two-decade-long development trajectory. Ethiopia is one of the fastest growing economies in the world and is embarking on its next phase of economic and social development supported by economic reforms and a bold programme of infrastructure investments.

### The Ongoing Transition

As a consequence of these limitations and due to rapid population growth^14^ (United Nations 2019) and uneven progress across the region, the number of energy-poor people is expected to remain unchanged in 2030 (IEA 2018) even in the best-case scenario where all current and announced government policies are implemented. In this context of under-achievement, although a shift to cleaner energy sources is partly taking place, talking of ‘transition’ may be an overstatement.

Some elements indicate nevertheless that many countries in sub-Saharan Africa are starting to move in the direction prospected by the International Energy Agency, that is, towards a power sector mostly fueled by renewable energy sources and natural gas.

In recent years, many African governments have designed energy strategies aimed at pursuing full energy access with the contribution of regionally abundant renewable energy sources. The number and size of wind and solar photovoltaic (PV) projects grew significantly, reaching a total installed capacity of 6,520 MW at the end of 2018, up from 535 MW in 2013 (Enerdata 2019). Such growth is the result of joint efforts between national governments, development finance institutions and private companies. Even when it comes to gas-to-power projects, many sub-Saharan countries are actively pursuing programmes to grow their gas economies, with gas-fired generation capacity doubled in the past few years, from 10.4 GW in 2010 to 20 GW in 2017 (Enerdata 2019). What Are then the Main Drivers Behind the Move of Sub-Saharan Governments Towards Cleaner Energy Sources?

#### Aligning to International Norms to Leverage International Support

One of the drivers of Africa’s move towards the energy transition is to abide by the Paris Agreement to which African countries are signatories. Despite sub-Saharan Africa’s negligible levels of CO_2_ emissions^15^ (World Bank 2018a), climate change will impact the continent more than many other geographic areas. This looming scenario makes the region a good candidate to receive ‘adaptation support’ provided by developed to developing countries in the framework of international climate negotiations.

Prior to the Paris Conference, African countries submitted NDCs outlining how they intended to address climate change and what they would do if adequate financing were available. They perceived COP21 as an opportunity to leverage support from the international community to achieve sustainable development (Africa Union 2015). Adding renewables to their power mix was indeed considered a way to obtain technical and financial support from development finance institutions mandated to support infrastructure enhancement in developing countries and, at the same time, attract investments and capacity building from international energy companies interested in gaining/increasing their foothold in African markets.

#### Reducing Costs for Energy Access

The second driver behind the move towards cleaner forms of energy is the reduction of costs to access energy. The fact that electricity tariffs often do not cover generation costs, hence putting utilities under financial stress, burdens state budgets and drives African governments to favour the use of cheaper generation sources. At the time of writing, the Levelized Cost Of Electricity (LCOE) of solar PV and wind technology has, in many cases, reached the fossil fuel range (IRENA 2019a, b, c).^16^ Furthermore, renewables have enabled the development of off-grid solutions that are increasingly attracting the interest of the most important international energy companies for their ability to provide energy access quickly and cheaply. According to Bilotta and Colantoni, ‘off-grid generation has a series of advantages for a large part of the African population, particularly the rural one, as it solves the issue of the dispersion of the population, requires smaller investments and is now affordable even for many of the most fragile consumers’. (2018, p. 4). However, while off-grid generation is, in many cases, the cheapest solution to ensure ‘basic’ energy access, it is instead largely insufficient to guarantee ‘industrial’ access, necessary to sustain the pace and size of economic development that the region needs.

#### Energy Security

Finally, the third driver of the low-carbon transition in Africa is energy security. Reliable supply of energy is one of the most important requirements for significant growth. African governments have started to identify the wind and the sun—abundant and widespread across the continent—as crucial sources of energy and means to increase energy security, through fuel diversification and greater energy independence (IRENA 2019a, b, c). Energy self-sufficiency reduces countries’ exposure to the price and supply volatility of importing energy.

### Case Studies

The cases of Kenya, Nigeria and South Africa represent different cases on how natural gas and renewable energy were introduced in the energy system to meet growing electricity demand. Different economic and political contexts in these countries shape the pursuit of the goal.

#### Kenya

Kenya is one of the most successful cases of renewable energy development in sub-Saharan Africa. Kenya’s electricity access rate increased massively from 15% in 2000 to 63% in 2017 (World Bank 2018b) and at the end of 2018 Africa’s largest wind power project—Lake Turkana Wind Power project (310 MW)—was completed. However, a slower-than-forecast economic growth led energy demand in the country to be incompatible with the numerous Power Purchase Agreements (PPAs) to which the government had previously committed. As a consequence, despite an incredible rise of renewable energy generation in the country, Kenya is currently facing difficulties in the reduction of electricity cost, hence halting the development of further renewable energy projects.

With a third of the population still lacking access to electricity, a major cause of missing demand is also linked to the current state of the power grid that does not allow power supply to reach final demand. Moreover, the high cost of power generation based on heavy fuel oil drove the government, in the past few years, to attempt shifting to natural gas (i.e. LNG). The shift, however, encountered resistance and did not gather sufficient political consensus. The introduction of natural gas interfered with plans to develop a 1050 MW coal power project.^17^

#### Nigeria

Nigeria is instead a prime example of infrastructure inadequacy and power system economic unsustainability. Despite having a total installed capacity of 12.5 GW and an energy access rate of 54.4% (World Bank 2018a), the country can deliver only about 5.2 GW of power to its citizens. The grid’s inefficiency and non-capillarity and the inability of the national utility to collect cash from its customers are the main causes of under-performance. This leads the government to use pollutants and costly diesel-fuelled generators as backup solutions to face frequent power shortages.

Also, political tensions add up as energy subsidies are the subject of an ongoing challenging negotiation between the government of Nigeria and the World Bank Group for a large financial package in support of the Power Sector Recovery Program.

#### South Africa

South Africa is a different case, and a peculiar one in the sub-Saharan context, both from an economic development perspective and an energy standpoint. The country’s GDP per capita (USD 6,339 in 2018) (World Bank 2019b) is far above the sub-Saharan average (USD 1,573); half of the electricity generated in sub-Saharan Africa is consumed in South Africa; 90% of generation comes from coal. However, the country suffers from power shortages, and its coal fleet is ageing.

The authorities planned a gas-to-power programme aimed at building and providing 3 GW of gas-fired plants. Gas will be both imported and domestically sourced. Though the government invited expressions of interest for the construction of a 600 MW gas power plant at either the port at Saldanha or at Richards Bay, at the end of 2017, the project was delayed. Later on, in September 2018, the government disclosed the Integrated Resource Plan (IRP) in which it outlined that gas-fired power was going to become more important in the power mix with LNG imports expected to drive the shift initially. However, according to Fulwood, political difficulties associated with the social implications of replacing coal with gas make the project unlikely to be developed before 2025 (2019, p. 16).

In terms of renewables, even though South Africa has been one of the first countries in Africa to develop solar and wind projects with the Renewable Energy Independent Power Procurement Programme (REIPPP), an ongoing political debate on the cost of energy is currently hindering the implementation of several renewable energy projects.

## Geopolitical Dynamics

Before deep diving into the geopolitical dimensions, it is worth reminding that the literature has so far ‘only barely scratched the surface with regard to exploring the potential geopolitical effects of the transition towards more renewable energy sources’ (Criekemans 2011, p. 4). Even less with regard to Africa.

In fact, though the energy transition has become a sensational topic of debate, much remains unclear about its geopolitical implications in Africa and globally. Predicting winners and losers in this transition then becomes for some highly uncertain (Hache 2016; Paltsev 2016), due to the increased complexity of energy geopolitics, consequential to a more heterogeneous set of technologies and actors involved. For others (Stang 2016; Huebner), it is more straightforward: winners are those countries with high energy consumption and few own resources like India, China, Mexico, Brazil and Europe. Losers are instead leading oil and gas exporters whose leverage decreases as energy types and suppliers diversify, and the need for long-distance transport of fuels diminishes due to decentral generation and smart grids.

In such blurred situation, a few analytical considerations are due. The first is that the arrival of renewable sources of energy is triggering a ‘transposition of the geopolitical logic of oil and gas onto renewables, despite the considerable differences between the energy types and their associated technologies and infrastructure’ (Overland 2019, p. 36). This chapter argues that while some dynamics inevitably remain unvaried, namely, dependence on external financing and technology, hence justifying the ‘transposition of the geopolitical logic’, the peculiar characteristics of renewables^18^ will add new dynamics to the geopolitical conundrum. In Africa, this mainly translates in a ‘regionalisation of energy relations’ (Scholten, p. 23).

Secondly, energy-related issues will partly be ‘less about locations and resources, and thus less geopolitical in nature’ (Overland 2019, p. 38). Rather, international energy competition ‘may shift from control over physical resources and their locations and transportation routes to technology and intellectual property rights’ (Overland 2019, p. 38). In other words, we may talk more about tech-politics than geo-politics. A caveat, however, is that natural resources still need to be extracted to produce technology, and Africa currently supplies some critical items. Access to natural ‘critical materials’ will hence be crucial and is likely to replicate the type of rent-seeking dependent relation with international actors typical of oil and gas.

These initial considerations need to be, however, contextualised in a world where the replacement of fossil fuels cannot realistically happen any time soon. Seemingly then, according to Scholten, ‘the fact that both fossil fuels and renewable energy will coexist in the energy mix for the foreseeable future implies that any (practically relevant) understanding of the geopolitics of renewables is in essence about how the energy transition affects fossil fuel dominated interstate energy relations’ (Scholten 2018, pp. 10–11).

### International Dependence and Regionalised Energy Systems

Even if Africa is becoming the locus for the extraction of ‘new’ resources needed to manufacture renewable energy technologies, as well as home to a variety of projects aimed at harnessing renewable energy, oil and gas are expected to be around for quite some time still.

On the one hand, there will be (there already are) endogenous drives to diversify economies and reduce reliance on hydrocarbons. On the other hand, global aspirations to increasingly use cleaner energy sources may translate into a decreased relevance of Africa’s role in global hydrocarbon fluxes, altering trade relationships and geopolitical connections. According to the IEA New Policies Scenario, global oil demand is expected to peak only in 2040 to then downturn. However, should the pace of the global energy transition unexpectedly accelerate,^19^ Sub-Saharan economies—in particular, those heavily reliant on oil revenues^20^—may be profoundly destabilised if diversification strategies are not timely implemented (IRENA, A New World The Geopolitics of the Energy Transformation 2019).

In this context, competition changes. While affordable *access* to oil, coal and gas is the main ground of competition in the fossil fuels world, in a low-carbon world, the struggle will still partly revolve around access to materials, required to manufacture technology, while also partly around how to *finance* infrastructure and control the *technology* needed to harness wind, solar and other renewable power sources. African countries will likely continue relying on external finance and technology to advance.

Nonetheless, these dynamics of dependence are increasingly complemented by dynamics of inter-dependency. While dependence characterises relations between Africa and international actors, interdependence is part of regional integration phenomena, in which the energy sector could act as a catalyst for development (see power pools).

#### Infrastructure Financing

According to Bilotta and Colantoni, ‘the SSA energy sector has suffered from the same chronic difficulty as African infrastructural projects in finding adequate investments, due to the lack of domestic funds as well as to a higher perceived regional risk’ (Bilotta and Colantoni 2018, p. 5). Domestic factors of instability, such as political struggles, GDP fluctuations, corruption and lack of transparency, currency risk, led African countries to mostly depend upon external actors to finance the development of energy projects. With renewables, the dynamic seems to be replicated.

Young African entrepreneurs in the energy space face challenges related to access to finance (in addition to lack of technical knowledge). Even though locally bred pay-as-you-go companies, such as M-KOPA and BBOXX, are rising, they face the challenge of having to raise sufficient capital to finance the upfront cost of solar panels due to reluctance from local financial institutions to provide financing. Hence, the need to rely on international investors, meaning that the inherent transaction costs, currency risks and profit expectations will be translated into higher solar home system prices, preventing greater uptake of decentralised off-grid solutions and limiting employment opportunities (IRENA, Renewable Energy and Jobs 2018, p. 23).

As for international investors, the incentives to invest in oil and gas infrastructure differ from those in renewables, including electrification. If in the oil and gas world, incentives are relatively high and resources can be sold on international markets, in the renewables-electric world, the non-exportability of resources and the need to use them in underdeveloped domestic markets make the incentive for the creation of power infrastructure lower. For this reason, domestic and international political support is crucial to guarantee the viability and long-term security of the projects.

Development Finance Institutions (DFIs)^21^ are increasingly playing an important role in supporting the energy transition in developing countries.^22^ In sub-Saharan Africa, DFIs are involved in infrastructure projects along the entire energy sector value chain. They make use of a varied range of instruments. In the last few years, the most important multilateral DFIs have been enhancing their support to renewable energy projects. Two of the most notable programmes supporting private renewable energy projects in sub-Saharan Africa are the World Bank’s Scaling Solar and the European Union’s External Investment Plan^23^—the former was launched in 2015, while the latter is more recent.

These two programmes provide technical and financial support to both governments and project developers for the development of power generation projects and mitigation of the off-taker risk through credit enhancement mechanisms (such as partial risk guarantee, which is a core instrument to make power generation projects bankable). However, while these programmes are effective in mobilising private investments in power generation, they are unable to address the deep causes of inefficiency and unsustainability of the sector. They need to be complemented by other initiatives along the electricity value chain for the power system to benefit effectively. Integrated approaches are hence preferred in this context.

The Temane Regional Electricity Project (TREP) in Southern Mozambique well exemplifies how an integrated approach looks like. In 2019, the Board of Directors of the World Bank approved a total USD 420 million of International Development Association (IDA) grants and guarantees to strengthen Mozambique’s transmission capacity for domestic and regional markets and increase electricity generation capacity. Developed as an integrated operation including both public and private investments, the TREP entails the construction of a 563-km high-voltage transmission line between Maputo and Temane and a private sector financing of a 400 MW CCGT generation plant in Temane. A USD 300 million grant will be provided for the construction of the transmission line and two IDA payment guarantees to de-risk the sale of electricity and the purchase of gas by the power plant. This approach requires a much more challenging and costly effort by all involved stakeholders. But the joint approach proves very effective as it simultaneously improves supply and demand.

In addition to centralised power generation, the World Bank has started also focusing on energy access programmes through mini-grid and off-grid projects so as to reach sections of the population that are not served by the national grid. This Africa-specific approach is crucial not only to bypass infrastructural deficiencies but also to ‘deliver energy in a way that African consumers will be able to afford—even the poorest strata of the population, and rural consumers in particular’. (Bilotta and Colantoni 2018, p. 6).

#### Control of Technology

The energy transition is above all about technology and innovation. African countries’ role in this realm does not differ, so far, from the role played in the oil and gas world. They are suppliers of critical materials needed to manufacture renewable energy technologies but do not hold the leadership in terms of technological innovation or control of technologies’ manufacturing. Hence, the relationship of dependence upon external actors is seemingly reproduced.

##### Suppliers of critical materials

The nascent energy transition and ever-evolving technological advancement have expanded the number of resources considered strategic. Africa is home to some so-defined critical materials, that is, materials ‘which most consumer countries are dependent on importing, and whose supply is dominated by one or a few producers’ (Overland, 2019, p. 37).

The Democratic Republic of the Congo (DRC), for instance, accounts for most of cobalt^24^ world production (more than 60%). Exports are mainly China-bound with the far eastern country being the world’s leading consumer in 2018. China primarily uses cobalt in the rechargeable battery industry. Securing access to the resource translated in the acquisition by Chinese companies of eight of the 14 largest cobalt mines in the DRC, accounting for almost half of the country’s output. South Africa is instead the world’s leading producer of manganese^25^ (30.6%) followed by Australia (17.2%), Gabon (12.8%), China (10%) and Brazil (6.7%).

Another strategic mineral present in Africa is Nickel. About 23.5% of reserves of this mineral are located in Madagascar and 4% in South Africa (USGS 2019). Other strategic materials such as lithium are overwhelmingly located in South America and are comparatively scarce in Africa in terms of reserves, resources and production.

It is important to stress that, while cobalt, nickel and other minor minerals are extracted from the African soil, their refining and manufacturing take place primarily abroad. In this process, China plays a fundamental role by virtue of its comparative advantage and remarkable refining capacity. In general, the extraction of these minerals presents a series of problems of environmental nature (particularly water-intensive and polluting). On top of that, the issues linked to the highly extractive mining industry (illegal mining activities, conflict subsidisation, low worker protection) have historically been taking a toll on local communities in Africa.

Nonetheless, though these materials are currently crucial, given the fast-pace of technological innovations in the field, it is not improbable that currently used materials may soon be displaced by others. In other words, their geopolitical relevance is vulnerable not because of their geological abundance/scarcity, but because of the rapidity of technological innovation.

##### Leadership in technological innovation

Advancements in renewable energy technology guarantee the centrality of a country in a wider geopolitical perspective as ‘innovation will be a key determinant of the pace of change’ (IRENA 2019, p. 27). Though it is clear that leadership in technological innovation mostly does not originate in Africa, Scholten notes that it is, however, unclear ‘how developments like great power rivalry between the US and China or the EU and Russia and technical innovations in batteries or ICT will influence the speed and direction of the energy transition and nature of energy systems’. (p. 4).

One way to evaluate countries’ approach to technological innovation is to look at how they fare in terms of patents. The foremost leader in terms of renewable energy innovation is by far China, with more than 150.000 patents as of 2016 (IRENA 2019). It is followed by the United States with a little more than 100.000 patents. Japan occupies the third place, soon tailed by the EU, where Germany is in a leading position with 30.000 patents.

As for Africa, the main owner of renewable energy patents is South Africa, with 63% of total African patents, followed by Egypt with 8% (UNEP, EPO 2013). Specifically, South Africa is the leader in the field of mitigation^26^ technologies, with 553 patents, accounting for 84.2% of the African total. Northern Africa—represented by Egypt (18 patents), Algeria (12) and Morocco (11)—follows suit, but lags significantly behind South Africa. Other realities, such as Ghana, Burundi, Mali, Senegal and Zimbabwe, have less than 1% of total African mitigation patents, with only Kenya reaching 1.2%. The rest of Africa possesses 3.9% of patents (25 over 657 in the entire African continent).

The number of African inventions related to renewable energy, as of 2016, was significantly lower than that found in other countries or regions, translating into higher exposure for African states to the shocks brought by the transition. Apart from the economic consequences of these shocks suffered especially by countries highly reliant on fossil fuels, lagging behind in terms of innovation corresponds to decreased political importance.

Nonetheless, African countries still possess a comparative advantage for other types of energy-transition-related technologies. These are Made-in-Africa technologies that facilitate access to energy. Examples are ‘smart payment’ or ‘pay-as-you-go’ systems. These smartphone-friendly apps are intended to facilitate payments in particularly remote rural areas where banks are not readily available.

#### Regionalised Energy Systems/Power Pools

The likely geopolitical implications of increased usage of renewables are first ‘a regionalisation of energy relations’ (Scholten, p. 23). Renewables intrinsically need to use electricity as a carrier and electricity is currently a regionally traded commodity—rather than internationally traded like oil and gas—due to long-distance losses (IRENA, A New World, p. 47). Second, they imply a ‘strategic emphasis on continuity of *service supply* instead of *commodity supply* due to renewables’ abundance and stringent managerial conditions’ (Scholten, p. 4). As a consequence, a shift to the green economy could increase intra-continental trade while creating important interdependencies.

This adds up to the existing geopolitical conundrums for two reasons. The first refers back to the first two points made about finance and technology. In order to trade electricity regionally, grids need to be improved, and this strongly depends on external finances and technology, which are not always readily available hence making electricity trade less quick or smooth than wished. Secondly, though grids’ development is needed in Africa, off-grid systems may develop more quickly and in a more widespread fashion, leading certain areas of the continent to leapfrog the grid system entirely.

Geopolitically, greater cross-border trade in electricity could create geopolitical vulnerabilities for electricity importers, but greater electric interconnection will also increase interdependence among nations, reducing risks of conflict. In fact, Overland challenges the fact that electricity disruptions can be used as a geopolitical weapon inasmuch as ‘much of the future international solar and wind power trade will likely involve more symmetrical relationships between different prosumer (producer-consumer) countries than does the unidirectional gas trade (and much past electricity trade)’ (2019, p. 38).

In a world in which energy can be produced in various locations, then it is less likely that few actors dominate the scene by controlling routes and chokepoints. However, control over grid infrastructure may become vital. While some argue that countries that dominate electricity grids may exercise undue control over their neighbours and that interstate electricity cut-offs will become an important foreign policy tool, applied strategically in the same way as oil and gas sanctions (O’Sullivan et al. 2017, as cited in IRENA), others note that ‘electricity trading tends to be more reciprocal than trade in oil and gas […] A country that generates solar power may import energy from a neighbouring country when it rains, but export to that neighbour when the sun shines’ (2018, 51). As a consequence, ‘renewable energy exporters will always be part of a complex web of interdependencies between importers and exporters that would tend to curtail the potential to use renewable electricity as a geopolitical weapon’ (IRENA 2018, p. 52). In Africa, however, this may be problematic given the levels of energy infrastructure development across the continent vary widely, potentially either making a country’s excess exports virtually impossible (for lack of electricity network or lack of payment capacity), or enhancing the asymmetry between energy exporters (countries with financial capacity to develop the production sector) and importers (countries unable to produce).

In sub-Saharan Africa, there are four power pools that have been ‘established to improve generation capacity and transmission infrastructure for greater cross-border trade and ultimately address a cost-effective way of evacuating excess capacity between countries to offset peak demands’ (Medinilla et al. 2019).

The Southern African Power Pool (SAPP) was the first electricity regional market created in sub-Saharan countries and it comprises 12 countries—nine of them are already interconnected; the East Africa Power Pool (EAPP) is composed of 11 countries but it is at a very early stage of development since it was established only in 2015; the West African Power Pool (WAPP), created in 1999 by the regional economic union ECOWAS, has ambitious development objectives with a USD 60 bn plan for transmission and distribution investments by 2030; the Central Africa Power Pool (CAPP), established in 2003 and comprising 10 member countries, is the least developed power pool in Africa and the one that would need the major infrastructure investments.

The vast disparities across countries in terms of political economies, governance and infrastructure could hinder efforts to create or further develop ‘grid communities’ or regional pools. Hence, strategic imperatives for success and growth of power pools in Africa require countries to focus on diversifying power generation sources by taking advantage of the renewable energy potential, which will help mitigate the respective power pools’ vulnerability and their impact on regional dynamics. Moreover, power pools will not reach their objectives if member state power utilities do not invest in transmission capacities and maintenance.

Moreover, the challenges to achieving functioning power pools are technical, but also political. Power pooling requires trust and a strong alignment of interests between the region’s member states, between the regions (and/or member states) and the national private sector, and between external partners’ and member states. Indeed, vulnerabilities (of domestic/regional systems) that hinder the development of regional energy integration could be mitigated if international actors contributed, pragmatically, to fostering ‘positive interdependence’ through support in developing priority transnational infrastructure.

In this context, it is worth noting that ‘increasing electrification of the energy system […] implies the reliance on a single transport modality’ (Scholten 2018, 23), hence replicating the same risky dynamic of lack of diversification. At the same time, this would certainly give to landlocked countries (generally disadvantaged in the oil & gas world) better chances of being connected and further their development goals.

### Socio-Economic Implications and Security Risks

Understanding the overall political and economic landscape, in other words, the particular context in which a transition is unfolding is crucial. Most accounts on the shift to renewables tend to see the energy transition as a mere shift in the energy mix from a source to another without accounting for the ‘disruptive potential of renewables to redefine energy systems and markets’ (Scholten 2018, p. 9) or for the fact that historic contingencies play a crucial role in technological change (Baker et al. 2014, p. 798). At the same time, it is equally important to identify the international macro-economic forces and political actors, institutions and processes that have an impact on how domestic policy choices and debates are shaped, enabled and constrained (Baker et al. 2014, p. 795).

#### Socio-Economic Growth and Disruptions

The transition to cleaner sources of energy can prove particularly disruptive in countries where fossil fuels provide crucial revenues to the state. While current importers of fossil fuels (or countries where production is for domestic use) are starting to replace fossil fuels for domestic use, many others in Africa are still heavily reliant on these sources. For oil, gas and coal producers, like Nigeria, Angola or South Africa, the decline in revenue generated from fossil fuel energy exports can provide an impetus for political reform and economic diversification. However, a decline in hydrocarbons revenue could also lead to political instability, especially in the short to medium term. These countries, unless they have ambitious strategies of economic diversification, could face some severe challenges in the near future. Indeed, countries in sub-Saharan Africa rank low on the Energy Transition Index designed by the World Economic Forum, signalling their lack of readiness for the energy transition.

However, renewables in Africa could also have a positive impact on economic growth. For instance, off-grid access to electricity can contribute to achieving better levels of ‘basic’ access across the continent—though ‘industrial’ access to energy will still be reliant on fossil fuels, hence constrained by the sector’s limits. Increased basic access, especially in rural areas, could slow down urbanisation processes, which in turn have a high impact on grid/electricity demand and on air quality (which has worsened significantly across the continent in the past 30 years^27^). Indeed, if citizens were guaranteed a decent and sustainable livelihood in rural areas, the impetus to move to the city might decrease. At the same time, according to IRENA the majority of countries in sub-Saharan Africa ‘will benefit from reducing fossil fuel imports and generating renewable energy domestically, because this will boost job creation and economic growth’ (IRENA 2019, p. 30), with the exception of Nigeria and Angola, whose dependence on fossil fuels rents will place them at risk.

##### Competing narratives and vested interests

The potential shift (even before becoming actual) translates into (often competing) narratives being played out, like the ‘energy security’ and ‘sustainability’ ones (Jacob 2017, pp. 348–349). Energy security narratives sustain that a country, to develop and industrialise, must be energy-secure and less vulnerable to disruptions to its energy supply. This approach justifies the use of whatever energy resource to support development. The case of South Africa is emblematic: considered by IRENA the leader in renewables development in Africa, the country is also Africa’s top coal producer, with the Mpumalanga Province being the second worst sulphur dioxide^28^ emission hotspot in the world (Greenpeace 2019). In Tanzania instead, the Minister of Energy and Minerals notes that ‘coal to electricity is necessary […] because its cost will be cheaper for citizens and this electricity will boost industrial growth’ (All Africa, 16 January 2016).

The counter-narrative implies instead a strict adherence to the global low-carbon movement. This approach sees clean energy as crucial to reduce dependence on fossil fuels and achieve sustainability and low carbon development. As Jacob notes in relation to Tanzania, ‘this narrative is grounded in the claim that ongoing and planned coal investments will create obstacles to Tanzania’s efforts to meet its obligations to reduce carbon emissions and mitigate climate change’ (p. 350).

Behind these narratives, Okem notes, there is a shift between ‘competing industrial sectors and political constituencies’ (Asuelime 2018, p. 3). The implications of the shift range from employment concerns, especially in those countries where fossil fuels employ large numbers of the population, to concerns over maintaining loyalty of voters and business circles for political survival (Whitfield et al. 2015). Indeed, if the energy transformation begins to permeate into industrial sectors that have traditionally been dominated by fossil fuel energy, there could potentially be severe social disruption, and we are currently seeing the fear of this disruption playing out in the coal industry.

Technological and political change is, however, also embedded and affected by broader global processes. In a study on South Africa, Baker, Newell and Phillips (p. 795) argue that global processes have the potential to alter the balance of power within the country between ‘entrenched coal-based interests’ and ‘emerging niches in renewable energy generation’ as both levels ‘interact with and are backed by’ international stakeholders. China, for example, has emerged as the ‘leading financier of Africa’s coal boom’ (Jacob 2017, p. 345).

##### Employment

Most African countries are expected to benefit, in terms of job creation, from the energy shift (IRENA 2019, p. 30). The sector’s workforce—in terms of direct, formal jobs—is already comparable to traditional power grids and utilities in Nigeria and Kenya where the job creation impact is expected to grow in the next few years—by 70% in Kenya and over 100% in Nigeria. However, these are exceptions. Employment of renewable energy still remains limited in Africa as a whole, especially when compared to the growth in Asia (60% growth in 2017 compared to 51% in 2013). Most of Asia’s dynamism is based on growing domestic deployment and strong manufacturing capabilities, supported by policies such as feed-in tariffs, auctions, preferential credit and land policies, and local content rules.

In Africa, instead, compared to the oil and gas sector—which is capital intensive but not labour intensive—or mining—which is very labour intensive but localised—employment in the renewables sector is less labour intensive (than oil and gas) but not comprehensively developed along the value chain. In fact, employment is generated especially in the sales and distribution, installation, and operations and maintenance of the supply chain—vis-à-vis manufacturing. As a consequence, as long as there is only a limited domestic capacity to assemble equipment or manufacture products, economic multipliers and the resulting employment and other benefits will accrue elsewhere.

South Africa is the country that employs the largest contingent of workers in the renewable energy sector, close to 35,000, distributed across solar PV, concentrated solar power (CSP) and wind. Important developments are also taking place in the off-grid sector. In Ghana, Africa’s largest solar PV project (Nzema plant, generating 155 MW) is likely to induce 2,100 local jobs through subcontracting and demand for goods and services.

However, a global review^29^ of skills for green jobs including four countries in Africa (Egypt, Mali, South Africa and Uganda) revealed the existence of a gap between the goals and targets set in environmental policies and the human resources available for their implementation. Skills gaps exist for technical and engineering positions and could grow as the renewable energy sector continues to expand, leading to project delays or cancellations, cost overruns and faulty installations. There are, however, promising experiences. For example, Cape Verde launched a Renewable Energy and Industrial Maintenance Center (Cermi), whose main activity is the training of professionals in the areas of design, assembly and maintenance of photovoltaic installations.

#### Security and Dominance/Cooperation and Conflict

Finally, renewables are likely to reduce conflicts as we know them, but other tensions are likely to arise around cybersecurity and access to important minerals (IRENA, ‘A new world’, p. 55).

In terms of cybersecurity, the fact that renewables rely on the use of electric grids as the carrier of energy, vis-à-vis pipelines, tankers, rail, road, the sea used to transport coal, gas and oil may reduce risks linked to crucial infrastructure or chokepoints. At the same time, however, grids may be subject to new vulnerabilities. Not because they are in Africa, nor because they are more likely to be cyber-attacked compared to other infrastructure (for instance, oil- and gas-related infrastructure). Rather because, as noted above, ‘increasing electrification of the energy system […] implies the reliance on a single transport modality’ (Scholten 2018, 23), hence replicating the same risky dynamic of lack of diversification. On the other hand, however, the diffusion of off-grid systems ‘may actually make the system more resilient, as many different units will have to be hacked to destabilize the system as a whole’ (Overland 2019, p. 38).

In terms of minerals instead, while cartels could develop around materials critical to renewable energy technologies, as noted above the fast-pace of technological innovations makes it likely for many of these materials to be displaced by others rather quickly, hence decreasing the chances of cartels—hard to form and sustain—to emerge (IRENA 2019, p. 54). In other words, their geopolitical relevance is vulnerable not because of their geological abundance/scarcity, but because of the rapidity of technological innovation.

## Conclusions

Any attempts to generalise sub-Saharan African dynamics, in the energy sector as much as in any other area, risk being over-simplistic given the diversity of structures, ideas, leaderships and international positioning across the sub-continent. While this chapter tried to present specific examples, it also made the effort to identify common traits so to provide a manageable framework to understand the energy transition in sub-Saharan Africa and its geopolitical implications.

Problematising the concept of ‘transition’ in African contexts, instead of taking for granted that a transition is well received and occurring, served the purpose of placing proper emphasis on Africa’s problem number one: access to energy, both ‘basic’ and ‘industrial’ access, that is, for households and the industry, respectively. Six out of ten people still lack basic access, and the remaining share is unable to consume as it would like due to non-reliable power networks or inadequate financial means. We then argue that most of Africa has very little to transit away from.

Nonetheless, for many governments, the idea of leapfrogging the conventional path to energy development and shift directly to clean energy is starting to become attractive. This is not only to win the hearts of the electorate—increasingly aware, if not victim, of climate change effects—but also to leverage the ad hoc financial support that international institutions are making available to help Africa achieve sustainable universal energy access.

Despite efforts, however, renewable energy—estimated by the IEA to be a crucial component, together with gas, of Africa’s low-carbon energy mix in the next decades—in sub-Saharan Africa represents today a mere 1% reality. This is due to the fact that although Africa remains an important supplier of natural resources (namely, oil and gas but also minerals to manufacture renewables technologies, as well as renewables), it continues to rely on external financial and technological support to develop energy systems. Nonetheless, the intrinsic features of gas and renewables are shaping the creation of regional energy markets. These will inevitably imply increased cooperation and coordination between neighbours across the region.

As a fact, looking at the energy transition in Africa will not merely imply monitoring the evolution of Africa’s dependence upon external actors, but will require a more alert acknowledgement that regional interdepencies are ever growing.

## Footnotes

1 By defining the nationality of a product, rules of origin dictate the conditions for the application of tariff concessions, delimiting the range of products eligible for preferential treatment (UNCTAD 2019, pp. 3-4).

2 The IEA 2018 Outlook uses the estimate of 600 million people without access referring to the year 2017, in which the overall population in Sub-Saharan Africa was 1.05 bn.

3 National Determined Contributions (NDCs) identify the post-2020 climate targets, including mitigation and adaptation, which countries committed to ratifying the Paris Agreement.

4 According to African Energy Live Data, as of August 2019, installed electricity capacity from wind and solar totals 411 MW in Kenya and 1565 MW in South Africa.

5 Primarily used in the residential and commercials sector where more than 70% of total biomass consumption is concentrated.

6 Energy mix and electricity mix data are referred to 2017.

7 Excluding from the analysis South Africa, Botswana and Zimbabwe, three countries mostly dependent on coal the power generation mix becomes hydro 51%, natural gas 26%, oil 18%, coal 1% and renewables 4%.

8 According to Enerdata, total gas production in sub-Saharan countries was 67.5 bcm in 2017 and local markets consumed only 32.8 bcm. Thus, more than half (51.4%) of the production was exported. In the last two decades, gas production in sub-Saharan Africa has constantly been above gas consumption and the level of net export in the coming years is expected to be growing due to new Liquefied Natural Gas (LNG) developments (e.g. Mozambique).

9 The revenue–expenditure gap of a public utility company is referred to as a ‘quasi-fiscal deficit’ because utility companies are usually state-owned and commercial losses often represent a government-sponsored collective subsidy.

10 Under a power purchase agreement between a power generation project and the national utility, the non-payment risk incurred by an independent power producer.

11 It can either be a gas pipeline or a regasification facility.

12 The six million people (21% of the population) still lacking energy access would need additional investment in the power grid or off-grid connection.

13 According to World Bank Data, in 2018, the GDP per capita level in Ghana was still less than 20% of global GDP per capita.

14 According to the United Nations, sub-Saharan Africa population will be 1.4 bn people in 2030 and will double in the next 30 years, reaching 2.1 bn people in 2050.

15 According to the World Bank, CO_2_ emissions in sub-Saharan Africa account for 2.3% of global CO_2_ emissions.

16 Irena reports that the decline between 2010 and 2018 in the global weighted average LCOE is 77% for solar PV and 35% for onshore wind. The global weighted average LCOE of solar PV was USD 0.056/kWh in 2018; the global weighted average LCOE of onshore wind was 0.085/kWh in 2018; the fossil-fuel-fired power generation cost range by country and fuel is estimated to be between USD 0.049 and USD 0.174/kWh.

17 Currently suspended by the National Environment Tribunal.

18 That is, being abundant and intermittent, relying on decentralised generation and usage of rare earth materials in clean tech equipment, mostly electric distribution, requirement of stringent managerial conditions and long-distance losses.

19 According to the Sustainable Development Scenario of the International Energy Agency, global oil demand falls to 93.9 mb/d in 2025 and 69.9 mb/d in 2040. The Sustainable Development Scenario provides ‘an integrated strategy to achieve energy access, air quality and climate goals, with all sectors and low-carbon technologies—including carbon capture, utilisation and storage—contributing to a broad transformation of global energy’.

20 For instance, according to the World Bank Data, fossil fuel rents amount to over 15% of GDP in Chad, South Sudan, Angola, Gabon, Equatorial Guinea and the Republic of the Congo.

21 Development finance institutions are legally independent and government-supported financial institutions with explicit official missions to promote public policy objectives.

22 According to the OECD, multilateral climate finance from developed countries to developing countries almost doubled in recent years, from USD 15.5 billion in 2013 to USD 27.5 billion in 2017.

23 The External Investment Plan has identified five priority areas, but we refer to the ‘Sustainable Energy and Sustainable Connectivity’ investment window.

24 Cobalt is a crucial component for the manufacture of lithium-ion batteries.

25 Manganese is another important material for batteries and, as of now, it has no satisfactory substitute in its major applications (U.S. Geological Survey, Mineral Commodity Summaries, February 2019).

26 ‘Climate Change Mitigation refers to efforts to reduce or prevent emission of greenhouse gases. Mitigation can mean using new technologies and renewable energies, making older equipment more energy efficient, or changing management practices or consumer behaviour. It can be as complex as a plan for a new city or as a simple as improvements to a cook stove design. Efforts underway around the world range from high-tech subway systems to bicycling paths and walkways’. (UNEP, Mitigation).

27 According to UNICEF, between 1990 and 2017 deaths from outdoor air pollution in Africa grew by 60% (2019, p. 4). The figure is expected to grow even further as population growth, industrial growth and consumption growth—all expected in sub-Saharan Africa in the next decades—increase levels of pollution, especially in urban areas.

28 Sulphur dioxide is a toxic pollutant that causes disastrous air pollution and premature deaths.

29 High-Level Political Forum on Sustainable Development. ‘Interlinkages between energy and jobs’. Policy Brief No. 13.
